# The Role of Nucleoprotein in Immunity to Human Negative-Stranded RNA Viruses—Not Just Another Brick in the Viral Nucleocapsid

**DOI:** 10.3390/v14030521

**Published:** 2022-03-03

**Authors:** Maja Šantak, Zrinka Matić

**Affiliations:** Department for Physical Chemistry, Ruđer Bošković Institute, Bijenička cesta 54, 10000 Zagreb, Croatia; zrinka.matic@irb.hr

**Keywords:** negative-stranded RNA viruses, nucleoprotein, T-cell immune response, B-cell immune response, vaccines

## Abstract

Negative-stranded RNA viruses (NSVs) are important human pathogens, including emerging and reemerging viruses that cause respiratory, hemorrhagic and other severe illnesses. Vaccine design traditionally relies on the viral surface glycoproteins. However, surface glycoproteins rarely elicit effective long-term immunity due to high variability. Therefore, an alternative approach is to include conserved structural proteins such as nucleoprotein (NP). NP is engaged in myriad processes in the viral life cycle: coating and protection of viral RNA, regulation of transcription/replication processes and induction of immunosuppression of the host. A broad heterosubtypic T-cellular protection was ascribed very early to this protein. In contrast, the understanding of the humoral immunity to NP is very limited in spite of the high titer of non-neutralizing NP-specific antibodies raised upon natural infection or immunization. In this review, the data with important implications for the understanding of the role of NP in the immune response to human NSVs are revisited. Major implications of the elicited T-cell immune responses to NP are evaluated, and the possible multiple mechanisms of the neglected humoral response to NP are discussed. The intention of this review is to remind that NP is a very promising target for the development of future vaccines.

## 1. Introduction

**Figure 1:**
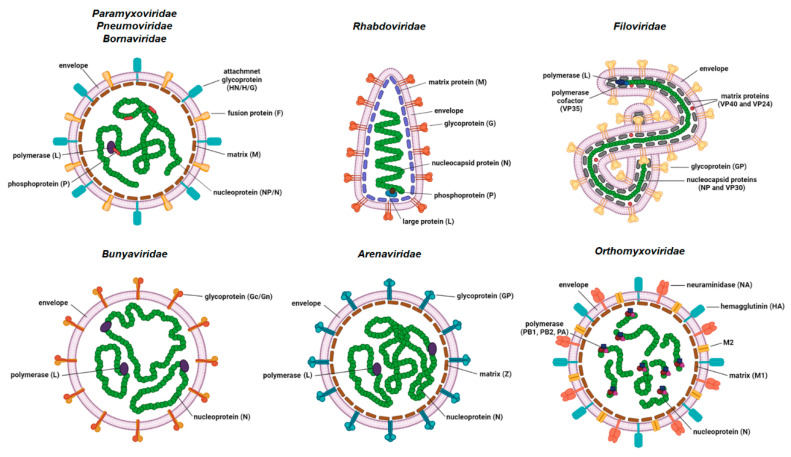
Basic schematic representation of the virion structures of the negative-stranded RNA viruses. Structural proteins are shown. Some genera-specific proteins are omitted from the representation for simplicity (created with BioRender.com, accessed on 21 December 2021).

Human negative-stranded RNA viruses (NSV) comprise nine viral families: *Paramyxoviridae* (measles virus, mumps virus and human parainfluenza viruses); *Pneumoviridae* (respiratory syncytial virus and human metapneumovirus); *Rhabdoviridae* (rabies virus); *Filoviridae* (Ebola and Marburg viruses); *Bornaviridae* (Borna disease viruses); *Orthomyxoviridae* (influenza viruses types A, B and C); *Bunyaviridae* (Rift valley fever virus); *Hantaviridae* (e.g., Puumala and Sin Nombre viruses) and *Arenaviridae* (Lassa virus and lymphocytic choriomeningitis virus (LCMV)). The genomes of NSVs consist of either non-segmented (ns) or segmented (s) single-stranded RNA. The former are members of the families *Paramyxoviridae*, *Pneumoviridae*, *Rhabdoviridae*, *Bornaviridae* and *Filoviridae*, while the later are members of families *Orthomyxoviridae*, *Bunyaviridae*, *Hantaviridae* and *Arenaviridae* (Figure 1). NSVs with a segmented genome have six to eight (*Orthomyxoviridae*), three (*Bunyaviridae* and *Hantaviridae*) or two (*Arenaviridae*) genome segments packed in a viral particle.

The size of the genome varies between approximately 8.9 kb for Borna disease virus and 19 kb for Ebola virus. The viral genome is tightly associated with multiple copies of nucleoprotein (NP)/nucleocapsid (N) protein, which form a highly ordered ribonucleocapsid (RNC) located inside the viral envelope. The viral envelope originates from the membrane of the previous host cell. RNC protects the viral genome from degradation and serves as a template for transcription and replication. The vast majority of other NSV structural proteins form a cohort of proteins that exhibit high functional analogy. RNA-dependent RNA polymerase (RdRp) attached to the RNC is mostly encoded by a single large gene. The exception is *Orthomyxoviridae*, where RdRp is a complex of three viral proteins, namely PB1, PB2 and PA. Some of the NSVs require an additional protein (polymerase cofactor or phosphoprotein (P)) for viral transcription and translation that is also attached to the RNC. The inner side of the viral envelope is coated with the matrix (M) protein in all NSVs except bunyaviruses. Glycoproteins protruding as spikes through the envelope are usually present in a multimer form (di-, tri-, tetra- or pentamers) and are the key proteins for viral attachment to the host cell and fusion of the viral envelope to the host membrane, which enables the entry into the host cell.

**Figure 2:**
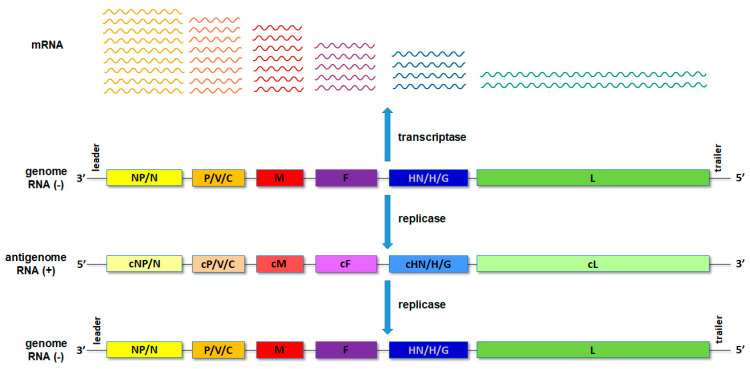
Schematic presentation of the transcription and translation processes with the RNA abundance gradient.

Upon entry of the virus into the new host cell, viral transcription initiates. This process is mediated by the orchestrated action of NP, RdRp and a cofactor/P. For the non-segmented NSVs, the viral proteins are expressed, sequentially generating an mRNA abundance gradient (Figure 2). This means that the mRNA of genes proximal to 3′ end of the genome will be produced in larger quantities than those located distally from the 3′ end [1,2]. In segmented NSVs, each genomic RNA segment contains a distinct transcription/replication complex (Figure 1). Arenaviruses have evolved a slightly different strategy than the rest of the NSVs. They use an ambisense coding strategy, and each of the two genomic segments directs the synthesis of two polypeptides in opposite orientations [3]. The replication of NSVs is a two-step process, since the genome cannot serve as a template for the synthesis of the new genome because of the same polarity. Thus, the replication starts with a generation of an intermediate antigenome, a full-length, faithful, positive-sense copy of the genome, and then a new genome is produced using the antigenome as the template.

NSVs globally cause a large number of infections, with symptoms ranging from a mild infection to life-threatening illness. Some of the world’s most prevalent respiratory diseases are caused by infection with NSVs such as influenza virus, respiratory syncytial virus (RSV) and human parainfluenza viruses (HPIV) types 1–4. In addition to respiratory symptoms, some NSVs can cause severe conditions such as lethal hemorrhagic fever (Ebola and Marburg viruses [4,5], bunyaviruses [6,7], hantaviruses [8] and arenaviruses (reviewed in Reference [9])) or manifest neuroinvasive potential, causing meningitis; encephalitis; myelitis and radiculopathy (measles virus, mumps viruses, rabies virus, arenaviruses [10], bornavirus (reviewed in Reference [11]), Rift valley fever virus [6,7] and arenaviruses [9]). While some of the NSVs inhabit exclusively humans (e.g., mumps and measles viruses), the others are zoonotic viruses (rabies virus, Ebola and Marburg viruses, Borna disease viruses, hantaviruses, Nipah virus and others) that use species other than humans as animal reservoirs and vectors (e.g., arthropods, bats and rodents) and occasionally infect humans upon transmission from the animal reservoir. Viruses causing zoonoses are far less contagious, because they do not have a strong capacity to spread from one human to another. Nevertheless, very often, zoonotic viruses cause severe illness and, consequently, have a high mortality rate. Zoonotic properties of the influenza virus stand out from other zoonotic NSVs, because the influenza virus not only has a high mutation rate (referred to as a genetic drift) but, also, the genome segments may reassort if two or more different influenza viruses coinfect the same organism (referred to as a genetic shift). These genetic modifications and changes can lead to adaptation of the new viral variant to a new host range and cause interspecies transmission (reviewed in Reference [12]). It has been shown that these events caused influenza pandemics in the 20th century [12,13]. Altogether, the NSVs have a global impact on health and economy, especially because they pose a continuous threat for outbreaks, epidemics and pandemics.

The therapeutic options against NSVs are limited, including vaccination as the most cost-effective strategy to reduce the mortality and morbidity caused by viral infections. A traditional vaccine approach has succeeded for measles and mumps viruses, so there are several effective live attenuated measles and mumps vaccines approved that are usually applied as a trivalent vaccine against measles, mumps and rubella. They were developed from wild-type isolates mostly in the 1960s [14,15,16,17,18] and have been in use for more than half a century. Meanwhile, pathogen evolution has continued generating new variants under the influence of vaccine-driven evolutionary pressure, and while measles and rubella vaccines seem to resist the evolutionary pressure, the mumps vaccine shows signs of failure. Despite the remarkable public health success of the mumps vaccine, evidence of virus escape from vaccine-induce immunity is piling up [19,20,21,22,23,24,25,26,27], suggesting that a new vaccine with better-matched epitopes or a different strategy might be needed soon. Although there were numerous trials to make a universal influenza vaccine (reviewed in Reference [28]), most influenza vaccines approved today still contain the inactivated virus, its subunits or purified hemagglutinin and neuraminidase of four influenza viruses (subtypes H1N1 and H3N2 of type A and lineages Victoria and Yamagata of type B) [29]. In contrast to measles and mumps vaccines, the influenza vaccine needs to be reformulated each season due to the genetic changes in the circulating influenza viruses [30]. Unfortunately, most efforts to make an effective human vaccine or treatment against other NSVs have been futile so far. Therefore, it is important to further analyze the pathogenesis of the NSVs and identify new potential viral targets for either effective vaccines or other drugs.

The development of new vaccines has been mostly focused on the importance of viral surface glycoproteins, because they are able to induce neutralization antibodies, which are considered to be the key determinants of vaccine-induced protective immunity. However, in viruses such as RSV, the antibodies to surface glycoproteins do not appear to be protective. Furthermore, viral surface glycoproteins have a high tendency to change their epitopes under selective pressure, and more conserved targets for the immune system are desired. NP is such a target, and the more we know about this protein, there is more and more evidence that this protein is much more than a structural “brick” hiding and protecting viral RNAs, both functionally and immunologically.

## 2. Structure and Function of NP

### 2.1. Genetic Stability of NP

**Figure 3:**
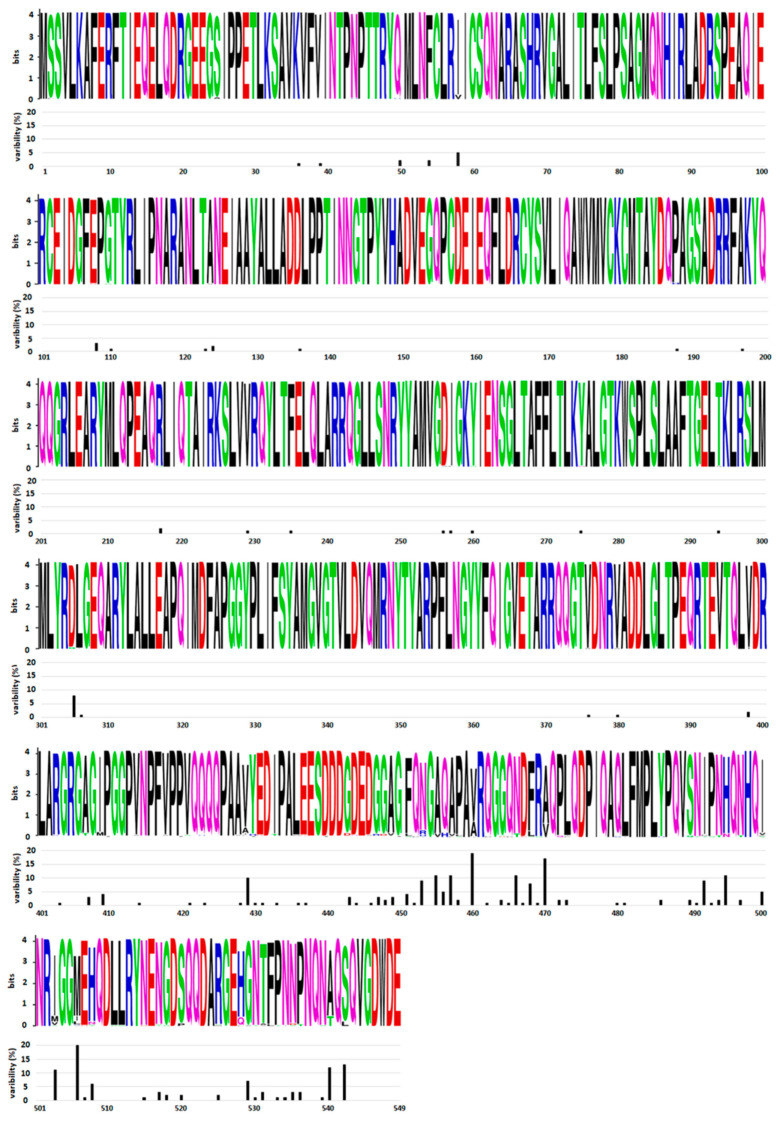
Distribution of amino acid substitutions in the mumps virus NP. Variability of the 82 mumps virus NP sequences from GenBank was analyzed. WebLogo was used to generate mumps virus nucleoprotein sequence logos (Crooks et al., 2004, https://weblogo.berkeley.edu/, accessed on 13 January 2022). Amino acid single letter annotation was used for each position in the sequence. The height of the column denotes the conservation of each amino acid position, while the height of the amino acid letter within the column indicates the relative frequency. Variability of each amino acid position is indicated below each logo panel.

The gene encoding NP is located at the 3′ end of the genome. Since NSVs have mRNA an abundance gradient (as mentioned above, Figure 2), NP is the most abundant viral protein in infected cells. The size of this protein varies between different NSVs and is in the range between 245 amino acids (aa) for Rift valley fever virus and 739 aa for Ebola virus. NP is highly conserved at the intraspecies level. A comparative analysis of 34 influenza NPs of viruses isolated from 1933 to 1990 showed that the average rate of amino acid substitutions is one amino acid every 6.6 amino acids [31]. A comparative analysis of all HPIV2 complete genomes from GenBank [32] also showed a high degree of similarity for *NP* genes (91.6%), as well as for proteins (93.4%). Both studies revealed that amino acid substitutions are not evenly distributed. The N-terminal part of NP is far more conserved (one aa substitution every 8.9 amino acids [31]), while the C-terminal part is prone to mutations (one amino acid substitution every 5.4 amino acid [31]). Further confirmation of the genetic stability and an uneven distribution of amino acid substitutions can be seen in Figure 3 for the mumps virus. The N-terminal part and central domain of mumps virus NP (aa 1–420) are highly conserved regions with only 16.1% substitutions in these regions, while the rest of the proteins, including the C-terminal N-tail (aa 420–549), is remarkably variable, with 83.9% substitutions found in this region only. As will be discussed in the next section on NP structure, the N-terminal domain of the NP is engaged in NP–RNA interactions and is densely packed. Thus, this region is neither exposed to the factors that would drive NP evolution nor can mutations be tolerated, as this might lead to a loss of viral fitness. In contrast, the C-terminal part does not participate in the NP–RNA complex and is a free N-tail domain. In this way, the N-tail domain is easily accessible to host factors such as enzymes and antibodies, which may generate a selective pressure and, finally, lead to increased variability. A limited NP conservation was also observed at the interspecies level in some members of the *Paramyxoviridae* and *Pneumoviridae* families. Cross-reactivity was tested with large panels of monoclonal antibodies against NP of the mumps virus, HPIV types 1–4, Sendai virus and Newcastle disease virus [33,34,35]. These studies show that the mumps virus and human parainfluenza virus types 2 and 4 are antigenically related viruses sharing some NP antigenically conserved sites. An antigenic similarity was also observed for NP of pneumoviruses RSV and HMPV [36]. Recombinant NP and whole virus lysates were reciprocally reactive with antisera prepared against either RSV or HMPV. Interestingly, a common immunoreactive site was found in the N–terminal region, aa 1–31.

Given the genetic stability of this protein, the attribute of a highly conserved protein paved the way for NP as a relevant biopharmaceutical.

### 2.2. Architecture of NP and Structural Organization of NP–RNA Complex

Since NP represents a potential antiviral target, a great amount of work was directed to gain more knowledge about the NP structure, the mechanisms of RNA binding, multimerization and RNC formation. NSVs pack their genome into RNC, a helical RNA–NP complex. The RNC is packed into virions. NP plays a key role in such an organized packing of the viral genome. Since the details of the NP structure and RNC assembly are beyond the scope of this review and comprehensive reviews have recently been published [37,38,39,40], only the basics will be presented and discussed, with a focus on those aspects especially related to further reading of this text.

New knowledge about the NP and RNC structures was first described for *Bornaviridae*, *Rhabdoviridae*, *Pneumoviridae* and *Paramyxoviridae*, followed by NP studies of the *Arenaviridae*, *Bunyaviridae*, *Orthomyxoviridae*, *Hantaviridae* and *Filoviridae* viral families [41]. The general principles and architecture of NP and organization of the NP–RNA complex have properties common to all NSVs, with some exceptions that may contradict this generalized comparison. Although the amino acid sequence homology is absent and substructural assemblies of NP monomers differ among different NSVs, related NSVs share highly conserved characteristics of NP folding, the binding of NP to RNA, etc. NP of all NSVs share the same domain organization. The NP protomer consists of two folded domains, a N-terminal core domain (NTD) and a C-terminal core domain (CTD). Even though there is a very low similarity in the primary sequence, all nsNSVs share a similar topology, having the RNA-binding site in between the NTD and CTD lobes, which form “jaws” that clamp the viral genomic/antigenomic RNA strand.

The main difference in the RNC between nsNSVs and sNSVs is its structure. The difference is explained by different RNC multimerization mechanisms. In nsNSVs, N- and C-terminal arms make identical interactions with neighbor molecules and, thus, form a helical structure, while, in sNSVs, flexible arms responsible for oligomerization form more irregular structures [42]. The RNA-free crystal structure of Borna disease virus NP revealed that NP assemble into homotetramers [43]. The authors calculated the potential mechanism of RNA binding. They suggested two models: (1) NP binds RNA through a positively charged central channel, or (2) RNA winds around each tetramer and forms linear RNPs. The second model offers a mechanism without NP disassembling to expose RNA. Although the structures of rabies virus NP and other NSVs are alike and possess similarities in folding, the place of RNA binding is different. Rabies virus nucleocapsid is oriented inside-out, with RNA inside and the protein outside, while other studied NSV nucleotide–protein complexes are the opposite [41,44,45,46,47].

Each NP protomer accommodates a fixed number of nucleotides. The number of accommodated nucleotides varies between viral families (e.g., six nucleotides per protomer (“the rule of six”) for paramyxoviruses [48] and nine nucleotides per protomer for vesicular stomatitis virus [49]). In the absence of viral RNA, NP binds any RNA available. To prevent this from happening, NP is chaperoned by P (or VP35 of the Ebola virus [50]), which maintains NP as a monomer and sequesters it from binding to any host RNA, thus preventing the formation of premature and empty virion. The assembly of the nucleocapsid occurs via the domain-swapping mechanism, where the NTD is essential while the CTD seems to be dispensable [40]. The very terminal region of the CTD, called the N-tail, is an intrinsically highly disordered region [51,52,53,54,55,56,57,58]. It is highly sensitive to proteolysis [52,57], and structural analyses of these regions are rarely successful [58,59,60]. To investigate the role of N-tail in RNC formation and facilitate the structural analyses of NP, multiple studies used recombinant NP, with the N-tail removed by trypsin. The removal of the disordered tail domain showed a more rigid nucleocapsid structure that could be more easily studied [46,61,62].

Recombinant NP spontaneously assembles into ring- or string-like structures. The crystal structure of a recombinant NP–RNA complex showed an organized structure containing a varying number of protomers: nine per ring for the rabies virus [63,64], ten per ring for RSV [65], 12–14 per ring for the measles virus [46,60,61,66], 13 per ring for the mumps virus [58,67], 11.8 to 15.8 per turn for the Ebola and Marburg viruses [68,69] and nine per ring for the influenza virus [44,70]. A mechanism of NP multimerization, by which the N-terminal arm of one subunit extends and interacts with the hydrophobic pocket of the next subunit, was proposed by the authors of Reference [71]. Assemblies of the Rift valley fever virus were more flexible than observed in nsNSV [72]. The study of the NP oligomerization in hantaviruses proposed an oligomerization mechanism in which the C-terminal arm of a protomer is inserted in the core domain of the following protomer [73].

Transitions among the different NP assembly forms must occur during the viral life cycle to be productive. They include: (i) a structure of the unassembled monomeric NP form bound in the complex with the phosphoprotein (complex N0–P), (ii) a structure that enables binding of the RNA and oligomerization of NP into helical RNC and (iii) a structure that releases RNA, making it accessible to RdRp. In addition to NP, P participates in orchestrated activities during these structural transitions. Unexpectedly, the N-tail is essential for regulating the structural transition from one assembly form to another by interacting with the disordered N-terminal region and the C-terminal X domain of P. The docking of the complex of P-RdRp during viral replication/transcription is also governed by its interaction with the N-tail [74], probably initiating the uncoiling of nucleocapsids [40].

### 2.3. Multiple Functions of NP

It was initially believed that the function of the most abundant viral protein was to protect viral RNA from degradation by cellular enzymes. Although this is true, it is only a part of the complete picture. Over the years, it has become clear that NP protects viral RNA, but it also shapes the helical structure of RNP and actively participates in and orchestrates the transcription and replication of viral RNA templates. Furthermore, different sets of functions, unrelated to the complex with viral RNA, relate to the ability of NP to induce immunosuppression in the infected cell or organism.

#### 2.3.1. Protective Function

NSVs have small genomes and are able to encode for few proteins. These few viral proteins are sufficient to ensure a new generation of virus only if they hijack accessory proteins of the host cell transcription/replication machinery. This means that the viral genome has to be in the cellular compartments (cytosol or, for the influenza virus, nucleus) where the cellular proteins are available throughout the whole viral life cycle. By doing so, the viral genome isconstantly in danger of being damaged or degraded by cellular RNases, which are normally present in these cellular compartments. Therefore, a physical separation away from the fatal host factors is an absolute prerequisite for a successful viral life cycle. The most obvious feature of the NP is the RNA-binding capacity. In fact, the NSV genomes are never free of NP in nature. For many NSVs, direct evidence on the RNA-binding mechanism has been proven by co-crystallization of the RNA–NP complexes [47,63,65,75,76,77]. Essential for the protective function of NP is its capacity to tightly bind the RNA and oligomerize and shape a helical RNC. The atomic structures described correspond to various oligomeric forms, except when the oligomerization domain was mutated [78].

Except from physical protection of the viral RNA, there is an additional impact of the tightly bound NP to RNA. Viral RNA, either in single-stranded or in double-stranded form, constitutes a very effective pathogen-associated molecular pattern (PAMP), which is a target for specific cellular pathogen recognition receptors (PRRs): membrane-associated TLRs (TLR3, TLR7 and TLR8) or cytoplasmic helicases RIG-I and MDA-5. By sequestering RNA from the harmful environment, the NP also prevents sensing of the viral RNA by PRRs and activation of downstream signaling, which would lead to the induction of interferon types I and III, interferon-stimulated genes (ISGs) and a number of proinflammatory cytokines (reviewed in References [79,80]). Thus, NP indirectly prevents immune recognition by the cells of the innate immune arm, which is critical for early detection and an antiviral response to invading viruses.

#### 2.3.2. Role in Transcription and Replication

NP is an important factor in regulating the replication and transcription of NSVs [37,81]. A seemingly indestructible assembly composed of viral RNA tightly packed within the shell of multiple NP copies might present a challenging constraint for processes that are about to take place upon the entrance of the virus into the host cell. Yet, a finely tuned and coordinated sequence of events will follow, leading to a highly successful transcription and replication resulting in a large number of progeny viral particles. During these events, NP transiently detaches from the ancestral genome to give the complex of viral RdRp and other viral and host cell cofactors access to initiate and carry out transcription or replication. A number of host cell cofactors have been found to have an important role in this process. Different RNA polymerase complexes (the transcriptase complex and replicase complex) were purified by the immunoaffinity method from vesicular stomatitis virus-infected baby hamster kidney cells [82]. The transcriptase complex consisted of virus-encoded RNA polymerase L and P proteins and cellular protein translation elongation factor-1alpha, heat-shock protein 60 and mRNA cap guanylyltransferase. On the other hand, the replicase complex contained viral proteins, L, P and NP but lacked elongation factor-1 alpha, heat-shock protein 60 and guanylyltransferase. It was shown that the RdRp of *Rhabdoviridae* and *Paramyxoviridae* preferentially performs transcription when NP is limited while favoring genome replication when NP levels are abundant [83,84]. In the absence of NP, the polymerase can initiate the synthesis of naked viral RNA, but its processivity is reduced [37,83,84,85]. Studies with the influenza virus have shown that NP is not required for the transcription and replication of short viral RNA templates up to 76 nucleotides in length but still supports the transcription of templates of up to 125 nucleotides at diminished levels [86]. This indicates that transcription and, subsequently, protein synthesis is a priority immediately after the virus infects the host cell. In contrast, as soon as sufficient amounts of NP that will protect and package the nascent genome are generated, genome replication can initiate, and the NP assembles with it, to form a new RNC. Thus, NP actively participate in and orchestrate these highly complex processes. By binding to the genome, the role of NP may correspond to the histones in eukaryotic cells. The NP of the influenza virus undergoes acetylation by two host acetyltransferases, GCN5 and P300/CBP-associated factor (PCAF), and this modification affects the viral polymerase activities [87]. Some studies demonstrated the direct association of the availability of free NP for binding to viral RNA or the suboptimal structure of NP with the excessive generation of defective viral genomes (DVGs). An insufficient level of NP was associated with an increased generation of DVGs, which, in turn, induced the host antiviral response in a RIG-I-dependent manner [88]. The same authors showed that this is applicable to a wide range of NSVs (Sendai virus, influenza virus type A, HPIV3, measles virus, vesicular stomatitis virus, Ebola virus and Lassa virus). Another study [89] suggested that only one substitution in NP can deoptimize the NP structure, which results in a lower density of the RNC, leading to a lower viral fitness, which, in turn, is critical for the integrity of Sendai virus RNC and results in the enhanced production of DVGs.

#### 2.3.3. Immunosuppression

As presented so far, NP has a protective role in physically separating viral genome RNA from the cellular components that may harm it, preventing PRR from being activated by viral PAMPs (i.e., viral RNA) and engaging in signaling employed in the induction of the proinflammatory response, and it precisely orchestrates transcription/replication and hijacks the cellular machinery. During this time, the host cell must be well-maintained, enabling adequate conditions for viral progeny to be generated. This includes preventing apoptosis and blocking immune responses towards viral components. NSVs have evolved different strategies to avoid immune mechanisms by the host. A transient but profound immunosuppression is one of them, with NP having an important immunosuppressive role for some NSVs, thus proving its versatility.

Infection with the measles virus is followed by acute and intense immunosuppression, followed by complications caused by opportunistic infections, resulting a high morbidity and mortality rate, especially in infants. The immunosuppressive effect of the measles virus is evident in T-cell arm inhibition [90], impaired proliferation of peripheral blood lymphocytes and lymphopenia [91], allospecific cytotoxicity [92] and an increased level of IL-4 with a decreased level of IL-2 and interferon (IFN) gamma [93]. Although immunosuppression by the measles virus involves multiple actions of different viral proteins, here, we evaluate only the role of NP in this complex interplay of viruses and the host immune system and the virus. Given that in situ hybridization showed that not even 1% of peripheral blood mononuclear cells are infected during the course of acute measles [94], such a profound and long-term immunosuppression would require an indirect mechanism. The measles virus NP protein in vitro studies indicate that the binding of recombinant NP to Fc gamma receptor type II (FcγRII) inhibits antibody production by human B cells [95] and impairs dendritic cell function [96]. Measles virus NP binds via its C-terminal part to the receptor for the Fc portion of immunoglobulin G, FcγRII/CD32 [96]. The intracellularly synthesized NP enters the late endocytic compartment, where it recruits its cellular ligand, the FcγR [97]. NP is then expressed at the surfaces of infected leukocytes associated with FcγR, and is secreted into the extracellular compartment, allowing its interaction with uninfected cells. Finally, cell-derived NP inhibits the secretion of IL-12 and the generation of the inflammatory reaction, both shown to be impaired during measles. Further, the mechanism of immunosuppression induced by measles virus NP was revealed by Laine et al. [98]. They identified a new receptor NR (nucleoprotein receptor) for extracellular measles virus NP released from apoptotic infected cells. NP binds to both constitutively expressed NR on a large spectrum of cells from different species and to human-activated T cells, leading to suppression of their proliferation. Laine et al. suggested that, after release in the extracellular compartment, NP binds to NR and thereby plays a role in MV-induced immunosuppression.

*Arenaviridae* also induce severe acute immunosuppression during infection. The NP has been implicated in suppression of the host innate immune system, but the mechanism was unknown until Hastie et al. showed that the C-terminal domain of the Lassa virus NP protein shows strong structural homology with DEDDh exonucleases [99]. Further characterization has shown that NP has a strict specificity for double-stranded RNA substrates. The exonuclease activity is essential for the ability of NP to suppress the translocation of IFN regulatory factor 3 (IRF-3) and block activation of the innate immune system [99]. Previously, it has been shown that LCMV NP directly binds to RIG-I and MDA-5 to restrict the regulation of the IFN type I response [100,101]. The same authors gave evidence that LCMV NP reduces IFN-β induction by blocking the translocation of IRF3 to the nucleus. Another mechanism of the immunosuppression in the family *Arenaviridae* was suggested by Russier et al. [102]. They suggested that Lassa virus NP is involved in the inhibition of antigen-presenting cell-mediated NK cell responses, which contribute to immunosuppression during Lassa virus infection. NP of the Junin and Lassa viruses suppress the host immune response by completely different means. Truncated forms of NP in these viruses inhibit apoptosis, acting as a decoy substrate for caspase cleavage [103].

The suppression of the IFN type I response at different steps of the IFN pathway has also been attributed to the NP of influenza viruses, the rabies virus and the Borna disease virus (BDV). Influenza virus NP exploits Hsp40 to inhibit the antiviral state in the host cell. It interacts with the P58(IPK)/Hsp40 complex, which causes P58(IPK) to dissociate from the Hsp40 complex and inhibit the phosphorylation of PKR, thus inhibiting PKR activation [104]. The findings on rabies virus NP [105,106,107] strongly suggest that rabies virus NP plays an important role in the evasion of innate immune responses in the brain—in particular, the evasion of interferon type I induction—and thereby in efficient propagation and spread of the virus, leading to lethal outcomes of infection. Studies in vitro have indicated that BDV NP inhibits the activation of IFN type I by preventing the nuclear localization of IRF7 and inhibiting endogenous IFN induction by poly(I:C), coxsackie virus B3 and IFN-β [108].

As presented above, the NP of different NSVs has evolved various means of immunosuppression during the arms race with the host. It can be expected that there are more means of immunosuppression than have been described so far.

Although multiple functions of NP have been described, there is no doubt that this multifunctional protein could prove in the future to be the number one viral protein when it comes to versatility.

## 3. Immunity to NP

Infections with some NSVs induce long-term immunity, preventing reinfections (e.g., measles and mumps), while multiple reinfections throughout life with others are common (e.g., RSV, HPIV 1–4 and influenza). Understanding of the processes during viral infection and immunopathology induced by the virus are some major aspects for the development of new vaccines and therapeutics. Exposure to an invading virus elicits an acute response by the immune system, which, in most cases, is able to control the virus spread and replication in the organism and its excretion, leading to resolving the disease. Virus-specific adaptive immunity is mediated by primary T- and B-cell responses and the generation of memory T and B cells. Memory cells function in the steady-state mode, awaiting the moment of activation upon the invasion of the matching pathogen. If or when it happens, they quickly respond to the secondary infection and are capable of faster and better control of the virus upon re-exposure, causing only mild symptoms or no symptoms at all. The vaccination paradigm is the generation of a long-term immunity against the pathogen-specific antigen(s) by developing effective memory T and B cells. Additionally, the vaccination should elicit a very similar dynamic of the immune response to one that develops upon resolving the natural infection and that can prevent reinfection. Neutralization antibodies (NAbs) are generated towards the viral surface glycoproteins essential for attachment to the host cells and fusion of the viral envelope to the cell membrane. If NAbs are present in sufficient titers and an adequate match between the NAb and antigen is achieved, they will be able to prevent reinfection. Therefore, the titers of NAbs produced by memory B cells are very often defined as correlates of protective immunity. A vaccine design that merely aims to induce memory B cells that will secrete virus-specific NAbs can be successful only if an adjuvant is used to generate a robust and long-term response. However, such a vaccine design may encounter an obstacle in the form of numerous mutations in viral surface glycoproteins, creating escape mutant viruses no longer susceptible to neutralization by the NAbs. Therefore, the vaccine design should ideally comprise the induction of a combination of both NAbs and CD8+ T cells. The NAbs response to NSVs is mostly well-characterized, but studies on T-cell responses and the role of non-neutralizing Abs (nonNAbs) are very limited. In order to gain more knowledge on how to include CD8+ T-cell responses and nonNAbs into vaccine designs for NSVs, more research will be needed.

Interestingly, the vast majority of NSV-specific antibodies are directed against NP, making this protein an immunodominant antigen. The internal localization of the NP diminishes the odds that these Abs could have any neutralization capacity. Indeed, the titer of NP-specific Abs could not be correlated with the neutralization titer of the total serum Abs upon infection or immunization. Nevertheless, such a high titer of Abs, the role of NP in T-cell induced immunity and a remarkable genetic stability, give this protein importance as a novel viral target. It can be considered as a potential vaccine candidate that may help to develop a broad protective vaccine.

We discuss here the role of NP in the specific induction of T- and B-cell responses and review the current knowledge relating to these issues. Obviously, the T and B cell responses should not be separated from each other, since they collaborate and complement each other during viral infection. However, for clarity, the T- and B-cell responses to NP will be presented herein one after another.

### 3.1. The Role of T-Cell Immune Response to NP

T cells are able to recognize antigens only if the specific epitopes are presented to the T-cell receptor (TCR) as a complex within the major histocompatibility complex (MHC), also known as human leukocyte antigen (HLA) (reviewed in Reference [109]). As mentioned above, immune responses mediated by T cells involve the primary response of effector expansion and differentiation ending with retreat but leaving memory T cells waiting for the secondary encounter with the antigen, which will ensure that the expansion and differentiation phases are triggered more rapidly. The major classes of effector T cells are CD4+ and CD8+ T cells. CD4+ cells become activated by antigen-presenting cells (APCs) expressing MHC class II–associated viral T-cell epitopes and co-stimulatory molecules. Although CD4+ T cells mostly act as T-helper (Th) cells, a small population of CD4+ cells display cytolytic activity [110]. Two different subsets of Th cells coordinate and promote either arm of the adaptive immune system. The differential activity of Th cells is based on the cytokine profile. Th1 cells produce mainly interferon gamma (IFN-γ) and IL-2 and activate predominantly the cellular response (cytotoxic CD8+ T cells (CTL), macrophages, etc.), while Th2 cells produce IL-4 and IL-13 and promote B-cell activation [111,112]. CD8+ T cells are proposed to play a major role in clearing from intracellular pathogens such as viruses. Upon infection, they are activated and migrate from the lymphoid tissue to the infection site. There, they recognize virus-specific epitopes associated with MHC class I molecules. These epitopes originate from viral proteins expressed de novo and processed intracellularly. Thus, CD8+ T cells distinguish infected from uninfected cells, selectively lysing virus-infected cells while uninfected cells are left undamaged. CD8+ T-cell lytic activity is mediated by two types of granules: perforin and granzymes. Perforin permeabilizes the membranes of the infected cells, enabling granzymes to enter the cells and induce apoptosis. CD8+ T-cell-specific epitopes are located on all viral proteins, both internal and surface. This enables a broad specificity displayed by these T cells. T-cell epitopes are much less prone to mutations as a result of the selective pressure. Hence, CD8+ T cells specific for conserved internal viral proteins, such as NP, can be exploited to improve vaccine strategies by attempting to elicit cross-protective immunity as well. Despite some apparent differences in the cytolytic machinery, it appears that the antiviral cytotoxic activity of CD4+ and CD8+ T cells is similar (reviewed in Reference [113]).

The T-cell arm of the immune response to NP and targeted vaccination to induce a T-cell response to NP sites have been extensively published, demonstrating that the NP-specific T-cell response—in particular, in cytotoxic T cells—is valuable for clearing up infections. However, due to the convenience of animal experiments, most were performed on mice. The comparative analyses of murine and human immunity in general and CD8+ T-cell repertoire to the influenza virus show that the findings from mice are not completely translationally equivalent to humans [114,115]. The murine repertoire to influenza virus A appears to be quite limited. In contrast, the human memory CD8+ T-cell response to influenza A virus is broadly directed to epitopes on a wide variety of proteins [114]. Hence, in this work, we will focus only on the findings of the T-cell immunity to NP obtained from human sources.

In some human NSVs, the role of NP in the T-cell response to infection has been better studied than in others. In fact, only a few NSVs have been looked at in detail (influenza virus, measles virus and Hantaan virus), while the NP-specific T-cell epitopes and the role of the epitope-specific T-cell response in most NSV infections still remain largely unexplored.

#### 3.1.1. Paramyxoviruses: Measles and Mumps Viruses

In spite of the massive use of the trivalent measles–mumps–rubella vaccine and, ever more often, measles and mumps outbreaks, the details of the cellular response to infections with these two paramyxoviruses are rather scarce. Proof that the cellular response is essential for the control of measles virus infection comes from patients with hypogammaglobulinemia that recover normally from measles. In contrast, patients with severe congenital or acquired anomalies in the cellular immune responses or combined deficiencies in the cellular and humoral responses develop a progressive disease with complications [116]. The immunosuppressive effects exhibited by the infectious measles virus have greatly hampered the in vitro study of the measles virus-specific cytotoxic response [117]. However, there is strong evidence that both HLA class I- and HLA class II-restricted T-cell responses play an immense role in the clearance of measles infection [118,119,120,121,122,123,124,125]. The study by Ilonen et al. showed that a specific cytotoxic response studied in seropositive adults was directed at NP and hemagglutinin [126]. Later, Jaye et al. indicated fusion protein as an additional target of the measles-specific T-cell response, along with NP and hemagglutinin [125]. Little is known about the nature of the NP epitopes recognized by CD8+ T cells. Moreover, some studies attributed the T-cell cytotoxic response to HLA class II-restricted T cells [121,122,123,127]. Few studies have identified several different CD4+ T-cell epitope regions (Table 1), although with a little controversy, because different reports have described the identification of mostly different CD4+ epitopes (Table 1). The foundation of this controversy may be in the fact that studies unraveling measles-virus specific CD4+ T-cell epitopes used indirect methods for the stimulation of isolated human T cells. Synthetic NP-derived peptides or whole NP were used instead of the infectious virus, which causes arrest in the T-cell cycle. Hickman et al. analyzed the proliferative response stimulated with synthetic NP-derived peptides 17–21 residues in length mediated by CD4+ T cells in association with HLA-DR antigens. Although all peptides were able to sporadically stimulate some of the donors, over 70% of all donors tested, which included both vaccinated and naturally infected donors, responded to NP peptides representing aa 271–290, 367–386, 400–420 and 483–502, suggesting that these peptides may be broadly recognized within an HLA-diverse population. The most frequently recognized T-cell epitopes in both naturally infected and vaccinated donors were located in the genetically heterogeneous carboxy-terminal half of N. Further analysis showed that some epitope regions were recognized by all naturally infected donors but by only a small portion of vaccinated donors and vice versa [128]. In contrast to these findings, Marttila et al. analyzed the CD4+ T-cell lines established from healthy controls and multiple sclerosis patients, all with a history of past measles infection. Their results were based on the T-cell lines created with the whole NP. Their work showed that the carboxy-terminal end of the polypeptide was not recognized by any of the tested T-cell lines [129]. The epitopes most often recognized by the T-cell lines were concentrated in two regions’ overlapping peptides (20 aa) containing aa 321–340 and 331–350 (Table 1). Other regions in the N-terminal part of NP were found only sporadically. The divergence of the results from Hickman et al. [128] and Marttila et al. [129] could be explained by the fact that Hickman et al. performed their study on T cells from donors recently revaccinated or infected, while Marttila et al. stated that the donors had a history of past measles virus infection. Additionally, a limited similarity between the CD4+ epitopes identified in vaccinated and naturally infected donors may be due to the differences of the measles virus strain used for vaccination and the strain that caused natural infection. Along with these studies, two additional studies identified two more CD4+ epitope regions: aa 185–199 [130] and aa 372–385 [131] (Table 1). It would be of interest to further investigate the induction of cellular immunity to the measles virus, especially in early life, as a foundation to the development of the measles vaccine that would circumvent problems such as the obstruction of effective vaccination by maternal antibodies in infants.

In the last two decades, numerous mumps outbreaks in highly vaccinated populations worldwide have been reported (reviewed in Reference [132]). By studying the contribution of cellular immunity to mumps vaccine failure, de Wit et al. identified the first naturally processed CD8+ T-cell epitopes of the mumps virus [133]. Of those epitopes, 5% (*n* = 2) were derived from NP. One epitope was located in the N-terminal part (aa 115–122, IPNARANL), while the second one was found in the C-tail (aa 504–512, GGMEHQDLL). Although such a low number of NP epitopes is somewhat surprising, the use of the Epstein–Barr virus-transformed B-lymphoblastoid cell line (BLCL) from a single donor as the antigen-presenting cells in the study by de Wit et al. could give a limited conclusion. However, it was an interesting finding of these authors that one of the confirmed NP epitopes (aa 115–122, IPNARANL) has been previously recognized as a CD4+ T-cell epitope as well (aa 110–124, GTYRLIPNARANLTA) [134]. The identified CD4+ T-cell clone expressed the activation marker CD137 and produced gamma interferon, tumor necrosis factor and IL-10 in an HLA-DR4-restricted manner upon peptide-specific stimulation. Moreover, similar to measles NP-specific CD4+ T cells, these mumps virus NP-specific CD4+ T cells exerted a cytotoxic phenotype and specifically killed cells presenting NP aa 110–124 [134]. The importance of this mumps virus epitope is yet to be confirmed. However, this conserved epitope may represent a unique peptide able to activate both arms of adaptive immunity. Thus, it may be of great interest in the development of an improved mumps vaccine.

#### 3.1.2. Pneumoviruses: RSV and HMPV

RSV and HMPV are two related pneumoviruses and major human pathogens causing a large number of acute infections of the lower respiratory tract with severe symptoms especially in infants, elderly and immunocompromised patients. Due to the unclear pathogenesis mechanisms of these two viruses, the need to administer these vaccines very early in life and the fiasco of formalin-inactivated RSV vaccine in the 1960s [135], there is currently no vaccine available, and the treatment is mostly only supportive.

Protective immunity generated following RSV infection seems to be impaired and short-lived, which allows multiple reinfections throughout life [136,137,138]. From the observation of individuals with functional T-cell deficiencies, it became obvious that adequate cellular immune response is required to stop virus shedding and to resolve the infection as has been shown for RSV [139,140,141]. In spite of countless efforts to decipher it, the mechanism underlying severe RSV infections is still largely unknown. Many studies, most of which were performed in murine model, indicate that severity of the RSV infections originates from Th2-biased lung pathology, while others associate them with an exuberant non-eosinophilic, lymphocytic, and neutrophilic response, or with a lack of inflammatory response [142,143]. The literature suggests that in the absence of the strongly polarized Th1 response, an imbalanced Th2 and Th17 response can prevail. NP has been demonstrated to be the major target antigens for cytotoxic immune response in man and mouse infected with RSV, whereas the G protein was not recognized and can at best represent a minor target antigen for CTL [144]. The dominant CD8+ T cell-specific epitope of NP was described to be aa 306–314 (NPKASLLSL) [145].

Although HMPV has been isolated relatively recently [146], undertaken studies have confirmed that HMPV-specific antibody response does not provide a complete protection and cannot effectively clear the virus as well [147,148]. A more comprehensive study of T cell immune response immunodominance hierarchy of all HMPV antigens was performed [149]. It showed that the immunodominant antigen for T cell immune response against HMPV is F followed by NP.

Given what is so far known about the immunity raised during RSV and HMPV infection indicates that RSV and HMPV evolved somewhat different modus operandi than other studied NSVs. The levels of NAbs targeting F and G proteins show correlation with resistance to reinfection, but protection is far from complete and lasts for short time only [137]. So, to ward off infection with these two viruses, a vaccine design would need an innovative approach based on alternative responses probably including NP as well.

#### 3.1.3. Hantaviruses: Hantaan Virus

T cell responses specific for Hantaan virus (HTNV) NP correlates with the reduction of the risk of progression to acute renal failure [150]. Humoral response and cytotoxic T cell response during infection with hantaviruses are mostly directed against immunodominant epitopes on the NP although other structural proteins are also involved [150]. An explanation for this is the fact that the NP represents the most abundant and most conserved viral protein expressed during infection [151]. First identification of cytotoxic T cell epitopes was performed by Van Epps et al. [152] (Table 2), who suggested that the infection with Hantaan virus results in the generation of cytotoxic T cell response to limited epitopes on the NP protein. In depth analysis by several later studies have identified more than ten additional HLA class I restricted epitopes in HNTV NP (Table 2). Some of the identified epitopes were found conserved among closely related hantaviruses such as Hantaan virus and Sin Nombre. Since NP-specific cytotoxic response is closely related with the progression and severity of the infection with hantaviruses, in particular hemorrhagic fever with renal syndrome (HFRS), and the fact that identified epitopes are conserved among different human hantaviruses, the NP could be a promising target for effective vaccine development against hantaviruses in humans.

#### 3.1.4. Filoviruses: Ebola Virus (EBOV)

Virus replication and infection with EBOV is believed to be largely controlled by T cell-mediated immune responses. The study of 32 Sierra Leonean EBOV disease survivors with confirmed clinical infections during the 2013–2016 West African outbreak showed that NP elicited the strongest and most abundant CD8+ T cell response [156]. Epitope mapping and HLA typing in these naturally infected individuals revealed that only minority of virus-specific CD8+ T cells were specific for GP, while the NP-specific CD8+ cells dominated. Epitopes were present throughout the NP but with a slight skew toward the N-terminal third of the protein. The same aggregation of CD8+ epitopes at the N terminal part of the NP was also described earlier by Sundar et al. [157]. They identified three HLA-A2-binding 9-mer peptides of EBOV NP using computer-assisted algorithm: aa 23–31, 56–64 and 74–82 (FLSFASLFL, RLMRTNFLI and KLTEAITAA, respectively). Interestingly, all three peptides were conserved in three different strains of Ebola (Zaire, Reston and Sudan). Sakabe et al. further suggested that a vaccine designed to elicit both humoral and cellular immunity should minimally include GP and NP as immunogens [156]. The recently approved Ebola vaccine contains both of them, as will be discussed in a later section.

#### 3.1.5. Arenaviruses: Lassa Virus (LASV)

Data from human studies indicate a critical and dominant role of T cells over antibodies in controlling and clearance of acute LASV infection and providing immunity to reinfection [158]. A delayed and weak NP antibody response after natural infection with LASV was detected only months after viremia has cleared and it seems to be strain specific [159,160]. Additionally, the treatment of Lassa fever infected patients with immune plasma does not lead to improved condition [161]. On the other hand, a strong memory CD4+ T-cell response against the LASV NP was reported in healthy Lassa-antibody seropositive as well as seronegative persons from an endemic region [162]. More recent study studied T cell response in human Lassa fever survivors and identified CD8+ T cell epitopes specific for NP at regions aa 155–164, 453–462 and 552–561 [163]. Data of Sullivan et al. suggest that LASV-CD8+ T cell responses can respond to antigens from other lineages to a high degree [164]. The antigenic regions that contributed to T cell response were identified within the Lassa virus NP and glycoprotein complex. Taken together, more knowledge of the epitopes recognized by T-cells will be needed for the development of a recombinant LASV vaccine effective across different LASV lineages.

#### 3.1.6. Orthomyxoviruses: Influenza Viruses

Influenza vaccines currently in use have some major imperfections: they provide only strain-specific protection, require annual update on the strain content and need to be administered yearly. New, universal vaccine which could elicit a broad and long-term protection against multiple influenza virus subtypes is desired. Regions conserved among different influenza strains and subtypes would be useful targets in achieving such cross-protecting vaccine.

It is well established that effective protective immunity against influenza virus infection is mediated by neutralizing antibodies, but the knowledge of the precise role of T cells in human influenza immunity is still incomplete [165]. The relevance and importance of the T cells in influenza infection was demonstrated by the study of 342 previously healthy adults in the H1N1 pandemic [166]. The pre-existing T cells to the pH1N1 virus and conserved core protein epitopes were correlated with clinical outcomes after incident pH1N1 infection. Higher frequencies of pre-existing T cells to conserved CD8+ epitopes inversely correlated with the illness severity. This was particularly pronounced within the functional CD8+ IFN-γ+ IL-2− population, cells with the CD45RA+ chemokine (C-C) receptor 7 (CCR7)− phenotype inversely correlated with symptom score and had lung-homing and cytotoxic potential. Furthermore, in the absence of neutralizing antibodies, CD8+ T cells specific to conserved viral proteins NP and M1 correlated with cross-protection against symptomatic influenza. This protective immune correlate could guide universal influenza vaccine development [166]. Furthermore, CD8+ T cells activated upon infection with seasonal influenza type A strains can cross-react with pH1N1 [167,168], highly pathogenic avian H5N1viruses [169,170] and H7N9 variants [171]. Similar finding was published by Wang et al. who showed that early effective CD8+ T cell response (most likely recalled from the memory pool) was associated with less cytokine/chemokine-driven inflammatory disease and better recovery of hospitalized patients with H7N9 [172]. Thus, early control of influenza infection by CD8+ T cells (and other cellular responses) is required to prevent exuberant inflammatory responses [173].

A study aimed at identifying a set of human T-cell epitopes that would provide broad coverage against different influenza virus strains and subtypes indicated PB1 as the major target for both CD4+ and CD8+ T cell response [174]. Other studies clearly indicated NP as a major target of immunodominant CD8+ T cell response during influenza type A response [175,176]. This discrepancy may reflect selection of donors, diverse infections and/or vaccination history of the donors or methodologies used in the studies. Whatever the reason for this discrepancy is, it is more than evident that highly conserved NP has very important role in T cell response elicited upon influenza infection or vaccination, and thus represents a good target for induction of broad type vaccine induced immunity. Due to the impact of this virus on public health and the continuous threat of pandemic it is ultimate interest to decipher influenza virus pathogenesis and immunity. The Immune Epitope Database and Analysis Resource (IEDB, www.iedb.org, accessed on 13 January 2022) has recorded 113 human CD8+ T cell epitopes and 163 human CD4+ T cell epitopes derived from influenza A NP. In comparison to those numbers, a systemic survey of the literature reveals relatively few well described human immunodominant CD8+ or CD4+ epitopes for NP (Table 3). As seen from Table 3, epitopes are evenly distributed throughout the whole NP sequence.

Townsend et al. published the initial CD8+ epitope for the influenza A virus NP to be in the region aa 335–349 [177]. Later on, Grant et al. in their study narrowed and finally defined a minimal epitope to be aa 338–346 (Table 3, [176]). Some of the other CD8+ epitopes in Table 3 are also reported in more than one publication, although not always as minimal epitope, potentially indicating that some epitopes are more dominant than others, e.g., epitopes aa 148–156 (TTYQRTRAL), aa 221–226 (RMCNIL), aa 338–346 (FELDRVLSF), aa 383–388 (SRYWAI). Table 3 also shows that identified CD8+ epitopes for influenza A NP have the potential of binding to a diverse set of HLA molecules. Thus, if used for a new universal influenza vaccine, NP would be able to provide a broad coverage of the human population, which is an ultimate goal for influenza vaccinologists.

Influenza B virus has recently diverged into two lineages Yamagata and Shangdong. The dominant CD8+ epitope for Yamagata lineage was identified in the region aa 166–174 (FSPIRITFL) [178]. The Shangdong lineage differs from the Victoria lineage in this epitope by having one amino acid difference at position aa 171 (FSPIRVTFL). The cross-protection against both lineages regardless of amino acid difference was conferred in mice [179], while the human confirmation of the cross-protective immunity is still pending.

Although influenza virus NP is highly conserved protein, a number of amino acid substitutions in this protein were associated with escape from human CD8+ T cells [180,181,182]. The mutations in epitope regions aa 380–388, 383–391, and 418–426 abolished class I-restricted presentation allowing escape from the CD8+ T cells recognition [181,182]. Epitopes aa 380–388 (SRYWAIRTR) and aa 383–391 (ELRSRYWAI) escaped from CD8+ T cell immunity by mutating position 384 (R384K and R384G). This mutation appeared in the 1993/1994 season by completely replacing previous variant. Immunodominant epitope aa 418–426 was identified to contain extensive variation among viruses from 1957, 1972 and 1980. These variations caused different degree of cross-reactivity including complete failure of CD8+ T cells specific for older variants to recognize more recent strains of influenza A [181].

Helper and cytotoxic CD4+ T cells in influenza virus infection play an extremely important role. Hence, several studies described mechanisms and specific epitopes for CD4+ T cells. Influenza-specific CD4+ cytotoxic T cells have been identified in seronegative human volunteers experimentally infected with either non-pH1N1 or H3N2 influenza viruses [183]. The preexisting baseline CD4+ T cell response, but not CD8+ T cell response, correlated inversely with illness severity and virus shedding following infection. The cytotoxic CD4+ response was primarily directed toward NP and M protein. These CD4+ cells also responded to pandemic H1N1 (A/CA/07/2009) peptides. The same authors suggested that these cells are an important statistical correlate of homotypic and heterotypic response and may limit severity of influenza infection by new strains in the absence of specific antibody responses.

A systemic study of Chen et al. of immunodominant CD4+ T cell responses to influenza A virus in healthy individuals showed that NP and M1 were immunodominant targets of CD4+ T cell responses as well [184]. They used in vitro expanded multi-specificity influenza A-specific T-cell lines and individual influenza A protein antigens produced by recombinant vaccinia viruses. Interestingly, the conservation of immunodominant epitope sequences correlated with an increased frequency of generated mutations, indicating a strong selective pressure within these prominent epitopes [184]. Further, immunoinformatic tools were used to identify predicted CD4+ T cell epitopes and predicted epitopes were confirmed both in HLA transgenic mice and with human peripheral blood lymphocytes [185] (Table 3).

Since cytotoxic CD4+ T cells accumulate with age and the CD4 cytolytic activity was found to be comparable between all age groups upon influenza vaccination [113], a science-based strategy for designing new vaccines for the elderly could be to focus on the NP or M1 peptides that carry cytotoxic CD4+-specific epitopes.

Although circulating influenza A strains are of diverse subtypes, an infection with one subtype may result with the effective protection against heterosubtypic virus [166,172,183,186,187,188,189]. Such cross-protective immunity can be attributable to the pre-existing immunity raised against inter-subtype conserved viral antigens. Since viral glycoproteins are main target of the genetic drift enabling virus to evade antibody response, cytotoxic response to more conserved influenza A virus proteins such as NP, M1 and polymerase proteins are thought to be major promoter of heterosubtypic immunity [187].

Several studies undoubtedly prove the in vivo existence of CD8+ cells which are able to provide cross-reaction with homo- and heterosubtypic variants of influenza A viruses [190,191,192,193,194,195]. Moreover, memory CD4+ and CD8+ T cells isolated from healthy donors with the history of seasonal influenza exhibited cross-recognition of at least one H5N1 internal protein [170]. M1 and NP were the immunodominant targets of cross-recognition demonstrated here. To go even further in details in the heterosubtypic protection, some of the epitopes identified and mentioned earlier (aa 199–207, 219–226, 225–233, 265–273 and 383–391; Table 3) elicited CD8+T cell responses in all donors they tested and were conserved between vaccine virus and Australian H1N1 and H3N2 isolates [170].

Conserved targets would be useful in formulating a ‘universal’ vaccine, as they would cover multiple viral subtypes. Such ‘universal’ vaccine design can potentially be addressed by a T-cell epitope ensemble vaccine comprising short, highly conserved, immunogenic peptides from influenza able to activate T cells. Generally, an NP based influenza vaccine oriented to generate cytotoxic T cell response could provide considerable breadth of protection against distinct influenza strains. However, it seems that the use of a single epitope might not be sufficient to reach protection across global population. It has been computed that for the population protection coverage (PPC) > 95% at least six epitopes are required for vaccine based on the T cell epitopes [196]. The calculation involves the cumulative phenotypic frequency of the relevant HLA alleles within the population restricting the T cell epitopes.

Although the extensive knowledge of influenza immunity/pathogenesis and different immunoinformatic tools are already available, a continuous evolutionary cat-and-mouse game between the virus and the host still makes a rational design of universal epitope-based influenza vaccine highly problematic.

**Table 3:** T-cell epitopes of influenza A virus NP.

Location	Sequence	HLA Antigen ^1^	Reference
37–54	GRFYIQMCTELKLSDYEG	A*01:01 A*02:01 B*08:01	[175]
39–47	FYIQMCTEL	A*24:02 B*15:09 C*07:02	[196]
67–76	RMVLSAFDER	A*03	[174]
91–99	KTGGPIYRR	A*11:01	[176]
139–156	WHSNLNDATYQRTRALVR	A*02:01 B*15:01 B*44:02	[175]
145–156	DATYQRTRALVR	A*68:01	[176]
148–156	TTYQRTRAL	A*02	[174]
158–166	GMDPRMCSL	A*02 A*02:03 A*24:02 B*08:01	[185,196]
172–181	LPRRSGAAGA	B*55:01	[176]
199–207	RGINDRNFW	B*57:01 B*15:13 B*57:02	[176,196]
217–234	IAYERMCNILKGKFQTAA	A*02:01 A*11:01 B*15:01	[175]
219–226	YERMCNIL	B*:18:01	[176]
221–230	RMCNILKGKF	B*44	[174]
225–233	ILKGKFQTA	B*08:01 A*02:02 A*02:03 A*02:06 A*02:09	[196]
251–260	AEIEDLIFLA	B*44	[174]
265–273	ILRGSVAHK	A*03:01 A*02:03 A*11.01 A*33:01 A*68:01	[176,182,185,196]
328–336	LVWMACHSA	A*02	[185]
329–339	QLVWMACHSAA	A*02	[174]
331–348	MACHSAAFELDRVLSFIK	A*02:01 A*24:02 B*12:02 B*35:03	[175]
335–349	SAAFEDLRVLSFIKG	n.d.	[177]
338–346	FEDLRVLSF	B*37:01	[174,176]
379–395	LELRSRYWAIRTRSGGN	A*01:01 A*02:01 B*08:01 B*07:02	[175]
380–388	ELRSRYWAI	B*08:01	[181]
383–391	SRYWAIRTR	B*27:05	[176,182]
397–414	NQQRASAGQISIQPTFSV	A*02:01 A*11:01 B*15:01 B*44:02	[175]
418–426	LPFEKSTVM	B*35:01	[180]
36–52	IGRFYIQMCTELKLNDY	DR1	[185]
51–68	DNEGRLIQNSLTIERMVL	DR1	[185]
75–89	RNKYLEEHPSAGKDP	DR1	[185]
113–130	KDEIRRIWRQANNGEDAT	DR1	[185]
147–154	TYQRTRAL	DRB5*01:01 DRB1*07:01 DRB1*11:01	[196]
204–218	RNFWRGENGRKTRSA	DR1	[185]
301–318	IDPFRLLQNSQVYSLIRP	DR1	[185]
310–327	SQVYSLIRPNENPAHKSQ	DR1	[185]
330–344	LVWMACHSAAFEDLR	DR	[174]
404–416	GQISIQPTFSVQR	DRB1*04:04	[184]
409–425	QPAFSVQRNLPFERVTI	DR1	[185]
463–475	VFELSDEKAASPI	DRB1*09:01	[184]

^1^ n.d.—not defined.

### 3.2. B-Cell Response to NP

The production of virus-specific Abs by B cells following antigenic stimulation is an important phase of the immune response to viral infection. While T cells are important for viral clearance, Abs act in different steps of the viral life cycle. They can prevent infection by virus neutralization, opsonization and virus inactivation, but can also facilitate destruction of infected cell. Additionally, the virus budding, spread and invading of virus from one cell to the neighboring cells can be prevented by Abs. All these actions are mainly directed towards surface glycoproteins of the virus as the most accessible targets. Viruses evade the Ab threat by mutating Ab-binding regions, shielding them by glycosylation, etc.

The predominant Abs raised during infection or vaccination with most, if not all, NSVs are directed against the NP. We can only speculate about the reason why such a high titer of NP-specific Abs is produced. The abundance of the NP in the virus and during infection might be the reason. However, the amount of the protein does not necessarily correlate with the level of specific Abs generated upon immunization. So more plausible explanation is combination of the great abundance of this protein and the inherent self-assembly property to form multimeric structure. Such a multimeric structure based on the repetitive units could effectively induce BCR crosslinking inducing cascade of molecular events in B cell signal transduction leading to enhanced germinal center B cell selection and differentiation into plasma cells finally resulting in Abs production [197]. This inherent property of NP to form a multimer structure has a potential to be widely used in the design of the vaccine based on the recombinant NP (rNP). As already mentioned, rNP assembles in homogenous rings composed of NP subunits [58,63,66], enclosing any available RNA representing a virus-like particles (VLP). A similar VLPs based on the L1 protein of human papilloma virus (HPV) have been effectively used as HPV vaccine (reviewed in Reference [198]).

The internal localization of the NP within the virion gives poor chance for the NP-specific Abs to have neutralization effect. That is the reason why these non-neutralizing Abs (nonNAbs) have been studied only sporadically in the past. In spite of that, protective effect of NP-specific Abs was proven albeit in animal studies. Vaccination with the influenza A rNP and passive transfer of non-neutralizing NP Abs fully protected against influenza A challenge in mice [199,200]. Although only poor T cell response was elicited, vaccination reduced the manifestation of disease symptoms and decreased the influenza virus titers in the lungs of the influenza-infected animals [199]. A recombinant BCG vaccine that expresses NP of RSV (rBCG-N-hRSV) protects mice against hRSV infection, eliciting humoral and cellular immune protection. Further, this vaccine was shown to be safe and immunogenic in human adult volunteers. In the model of neonatal calves, this type of immunization increased virus-specific IgA and virus-neutralization activity in nasal fluid and increased the proliferation of virus- and BCG-specific CD4+ and CD8+ T cells in lymph nodes supporting the notion that this vaccine approach could be considered as a candidate for infant immunization against RSV [201]. RSV may induce an inappropriate Th2-type immune response, which causes severe pulmonary inflammation. The immunization of BALB/c neonates, which are highly sensitive to immunopathological Th2 imprinting, with rNP and adjuvants shows that protective vaccination against RSV can be achieved in neonates but requires an appropriate combination of adjuvants to prevent harmful Th2 imprinting [202,203]. Like in most NSVs, in hantavirus infection NP represents a major target antigen. At the very beginning of the acute phase, IgM and IgG antibodies can be detected that react with hantaviral NP [204,205,206,207,208,209,210,211] while antibodies against Gc and Gn appear later during the progress of disease [212]. Furthermore, the serological profile following Hantaan virus infection (hemorrhagic fever with renal syndrome (HFRS)) inversely correlates with the level of NP-specific Abs and illness severity [213].

Since immune system is a highly complex and more and more new mechanisms and pathways are being discovered every day, it is clear that there is more beyond NAbs and that nonNAbs are somehow involved in the humoral response to viral infection. To be accessible for a specific Ab and for them to be able to act, NP must be located on the surface of the infected cell at least briefly during viral life cycle.

Although the classical model implied that only surface glycoproteins of NSVs are present on the surface of the infected cells, a number of studies have shown that at least some NSVs (e.g., RSV, LCMV, influenza virus, measles virus and mumps virus) contradict this model. The first examples of an NSV whose nucleoprotein have been directly demonstrated on the surfaces of both the infectious virus and the infected cell came from the research of Fernie et al. [214] and Zeller et al. [215]. Fernie et al. showed that RSV NP can be detected on the surface of continuous BALB/c mice embryo cell persistently infected with RSV [214]. Several years later Zeller et al. demonstrated that NP of LCMV can be detected on the cell surface of the infected chick embryo cells in vitro [215]. It has been shown that influenza virus NP is expressed on the surface of infected cells for some time and, therefore, can serve as a target for antibody-dependent immune mechanisms [216,217,218,219,220,221]. Influenza virus NP was detected in the airways of infected mice as early as 2–3 d post-infection, it was still present at day 7, and had declined to undetectable levels by day 9, corresponding with the typical time of virus clearance [200]. Soluble NP was detected in nasal washes [200], supernatants of infected MDCK cells in culture and also on the surface of influenza-infected cells in vitro, along with barely detectable levels of M1 [216,217,220]. Mumps virus NP can be detected at the surface of Vero and A549 cells infected in vitro (Šantak, unpublished results). The surface expression of mumps virus NP is Golgi dependent and can be inhibited by brefeldin A. Additionally, the cycloheximide experiments reveal that mumps virus NP on the surface originated from de novo synthesized protein. Although all these findings provide indisputable evidence of the presence of the NP at the surface of infected cell or the secretion of the soluble protein in the cellular environment, almost nothing is known about the mechanism by which NP is transferred to the cell surface or out of the cell.

Given that NP is found at the cell surface, the nonNAbs are able to exert their protective function through immune mechanisms such as antibody-dependent cellular cytotoxicity (ADCC), antibody-dependent cellular phagocytosis (ADCP), antibody-mediated complement-dependent cytotoxicity (CDC), antibody-dependent intracellular neutralization (ADIN) and antibody-mediated inhibition of formation of new viral particles. Multiple mechanisms could involve NP specific nonNAbs at the same time, though neither of them has been studied in detail.

ADCC is suggested by a couple of studies to be the main mechanism of action of influenza NP-specific nonNAbs. Immunization of mice with rNP elicited NP-specific IgG which promoted influenza virus clearance in mice by using a mechanism involving both FcRs and CD8+ cells. Furthermore, anti-NP IgG rescued poor heterosubtypic immunity in B cell-deficient mice, correlating with enhanced NP-specific CD8+ T cell responses [200]. Another study shows variable results for influenza NP-specific ADCC activity. It provides strong evidence that non-neutralizing NP-specific Abs could play an important role in ADCC by being able to opsonize NP, bind dimeric rsFcγRIIIa and mediate NK cell activation. The NK cell activating IgG was found in human sera and IVIG [222]. However, when using cells infected with recombinant vaccinia viruses exclusively expressing NP as a surrogate for influenza-infected cells enhanced cytotoxicity could not be observed. The authors suggest that opsonizing antibodies to NP and M1 likely contribute to an antiviral microenvironment by stimulating innate immune cells to secrete cytokines early in infection. In particular, NP-specific nonNAbs would form circulating immune complexes (ICs) by opsonizing soluble NP. The ICs trigger activation of NK cells and innate immune cells to release pro-inflammatory cytokines and chemokines capable of activating and recruiting different effector cell types (macrophages, neutrophils and T-cells) [222].

Innate signaling pathways generate an antiviral state by producing proinflammatory cytokines, such as NF-kB, AP-1, IRF3, IRF5 and IRF7. The same transcription factor pathways were shown to be triggered by ADIN as well [223]. ADIN activity has not been shown to be the property of NP-specific Abs, but it has been shown for nonNAbs specific for P and M proteins of measles virus. P-specific IgA prevents the virus from evading type I IFN signaling [224] and blocks P-NP interactions, which decrease the synthesis of viral genome RNA and mRNA [225]. M-specific IgA mAb was able to effectively inhibit viral replication by ADIN up to 78% [226]. ADIN mechanism was well studied in rotavirus, a double-stranded RNA virus of the family Reoviridae. Rotavirus inner core protein VP6 and NP share some general features: both are highly conserved inner proteins, very abundant and the most immunogenic proteins in naturally infected humans. Rotavirus VP6 protein also participates in the viral capsid formation and self assembles in different nanostructures [227,228]. Intracellular neutralization of conserved inner core protein VP6 of rotavirus by VP6-specific IgA was shown to act by interfering with the viral replication cycle, in particular by inhibiting transcriptase activity [229,230]. The equivalent activity could be possibly applied to NP-specific IgA or IgG. The study of Bai et al. suggest that IgG could have ADIN activity albeit under certain conditions. An intracellular neutralizing activity for an influenza hemagglutinin-specific monoclonal IgG Y8, which has neutralizing activity only at an acidic pH was detected when Y8 was applied to the basolateral surface of MDCK cells expressing the rat neonatal Fc receptor for IgG (FcRn). Viral replication was significantly reduced following apical exposure of the MDCK cell monolayer to influenza virus. Prophylactic administration of Y8 mAb before viral challenge in WT mice, but not FcRn-KO mice, conferred protection from lethality, prevented weight loss, resulted in a significant reduction in pulmonary virus titers, and largely reduced virus-induced lung pathology [231]. Similar concept could work for NP whether it is present in the cell, as a soluble form outside the cell or on the surface of an infected cell. The FcRn express, among others, cells derived from bone marrow, mainly antigen presenting cell. The formation of the IgG-NP immune complex (IC) could involve FcRn to transport the IC to the degradative compartments involved in antigen presentation (reviewed in Reference [232]). Taken together, this implicates that NP-specific Abs could facilitate immune response by transcytosis-mediated mechanism although this is highly speculative, and the scientific evidence is required.

A different model of action than models described above is probably involved in the findings of Straub et al. They studied the mechanism of LCMV-specific IgG Abs isolated from LCMV immune serum. These Abs were mainly directed against the viral NP and completely lacked virus neutralizing activity, but accelerated virus elimination. Moreover, mAbs specific for the LCMV NP were also able to decrease viral titers after transfer into infected hosts. Intriguingly, neither C3 nor Fcγ receptors were required for the antiviral activity of the transferred Abs [233].

Antibody-mediated inhibition of formation of new viral particles is one more mechanism of how Abs can reduce viral burden and enhance viral clearance. The experiments with measles virus and human convalescent serum provide evidence that multivalent antibody can redistribute measles virus antigens on the surface of infected HeLa cells in culture causing capping, a clustering of viral proteins on one pole of the cells. The formed caps are being most likely extruded into the cell culture supernatant and disintegrated, thus decreasing virus production [142,234]. This mechanism seems to be working for glycoprotein-specific Abs of Marburg virus. NonNAbs against glycoprotein of Marburg virus drastically reduced the budding and release of progeny viruses from infected cells and inhibited the formation of virus-like particles (VLPs) [235]. The addition of NP-specific mAb to mumps virus infected Vero cells resulted in a decreased virus titer, attributing this observation to inhibition of new mumps virus particles formation similar to that described for measles and Ebola viruses (Šantak, unpublished data).

Clinical progression of NSV’s infection due to Fcγ receptor-mediated antibody-dependent enhancement (ADE) has not been described so far. So, the possible concern about the potential ADE of infection mediated by NP-specific nonNAbs should not be neglected, but does not seem to be very likely.

Collective lack of interest in humoral response to NP during NSVs’ infection and nonexistence of standard methods to measure it (both qualitatively and quantitatively) is responsible for the fact that very little is known about the role and the mechanism of NP-specific Abs activity. Additionally, there are almost no data on the position of epitopes for this immunodominant viral protein. A panel of six monoclonal antibodies against NP of HPIV1 and a series of truncated NP was used to get more insight into a topology of this protein. As a result, they discovered that half of the tested antibodies were specific for the last 23% of the C-terminal of the NP. Additionally, they showed that two out of six mAbs showed cross-reactivity with Sendai virus, a related murine parainfluenza type 1, indicating that these two epitopes are conserved between these two related viral species [236]. Our unpublished observation with the overlapping 30 amino acid long peptides of mumps virus NP and polyclonal sera obtained from rabbits and rats immunized with whole mumps virus indicate the localization of linear NP epitopes within the distal C-terminal region spanning aa 415–430, 440–465 and 495–520. The location of epitopes at the N-tail domain of these two viruses makes sense since the N-tail is intrinsically disordered and exposed at the surface of the nucleocapsid. Thus, it is easily accessible for specific Ab to bind. As discussed earlier, this region is also proved to be more variable than the rest of the NP, probably as a result of the Ab-driven evolutionary escape.

As presented here, in spite of the high interest and need to invent vaccines which will be able to generate a broad cross-protective immunity by the use of highly conserved internal viral proteins such as NP, humoral immunity to NP remains sporadically investigated and thus largely uncharacterized.

## 4. Vaccines Based on NP

Protection against viral glycoproteins is mostly strain specific due to the high variability of antigenic sites which are under selective pressure by specific Abs. Broader immunity can be achieved by cell-mediated immune response and, at least partly, by humoral immunity as well, elicited to the epitopes of more conserved structural proteins such as NP and M protein. When testing new vaccine candidates, the main focus is their immunogenicity and safety. The testing results should provide relevant information on the quality and quantity of the immune response (both humoral and cell mediated) and the safety of the tested compound [198]. Standard methods for measuring cellular response or the level and quality of NAbs are well known. However, to test the effectiveness of humoral response, which is mediated by nonNAbs, new assays should be standardized or even developed. Since nonNAbs may act through several different mechanisms, and even through mechanisms uncharacterized so far, this is far more complex.

Different novel technologies have been used for the design of NP-based vaccines: recombinant NP (influenza virus, HMPV, hantavirus, rabies virus), peptides (Ebola virus, rabies virus), DNA (influenza virus, Ebola virus, LCMV, Lassa fever virus, rabies virus), virus-like particles (RSV), Bacillus Calmette–Guérin (BCG) as vector (RSV) and various viral vectors (modified vaccinia Ankara (MVA) vector for influenza virus, Ebola virus and Lassa fever virus, rabies virus; measles virus vector for Lassa fever virus; poxvirus vector for rabies and CMV vector for Ebola). The number of published animal studies describing promising NP-based vaccine candidates (mostly in a murine model) is too numerous to discuss them and cite them all. Despite such a high number of published animal studies on potential vaccine candidates, only few have entered clinical trial procedures (Table 4).

### 4.1. Vaccine Candidates Based on the NP as Antigen in Clinical Trials

The European Medicines Agency (EMA, https://www.clinicaltrialsregister.eu/, accessed on 3 February 2022) and the Food and Drug Administration (FDA, https://clinicaltrials.gov/, accessed on 1 February 2022) were surveyed for vaccine candidates in clinical trials phase 1–3 based completely or partially on the NP for any of the NSVs (Table 4). Seven vaccine candidates were found, most of them in the status of completed either phase 1 or phase 2. It is anticipated that at least few of them will enter next phase. Four of them are intended for influenza vaccine: MVA-NP + M1 (University of Oxford), combination of MVA-NP + M1 and seasonal inactivated vaccine (Vaccitech Limited), ChAdOx1 NP + M1 (University of Oxford) and OVX836 (Osivax). Additionally, three vaccine candidates are intended for vaccination against RSV infection: MVA-BN-RSV (Bavarian Nordic), ChAd155-RSV (GlaxoSmithKline) and rBCG-N-hRSV (UC Chile). Interestingly, only one of the candidates is based on the recombinant protein (OVX836), while others are based on viral vectors (MVA or ChAd) or BCG as vector.

#### 4.1.1. Influenza Vaccine Candidates Based on the NP

Influenza vaccines used nowadays are mostly inactivated influenza virus or derivates of it, i.e., surface proteins hemagglutinin and neuraminidase. Far less frequently used are influenza vaccines based on the live attenuated influenza virus. Major disadvantage of the current influenza vaccines is the need for yearly update of the vaccine strains due to the genetic drift of the circulating viruses which mostly affect highly polymorphic viral surface glycoproteins. This also means that the immune response to such vaccine is limited to strain-specific Abs and the vaccine should be received each year. The ultimate goal for influenza vaccine development is universal vaccine which would elicit a long term and broad heterosubtypic immunity. Such a vaccine cannot be achieved by eliciting immunity solely to surface glycoproteins, but by using more conserved internal proteins such as NP and M1 protein (Table 4).

MVA-NP + M1 is a recombinant, replication-deficient MVA vector expressing the influenza antigens NP and M1 as a fusion protein [237]. Several Phase I and Phase IIa trials using MVA-NP + M1 generated in either chicken embryo fibroblast cells or in the duck immortalized AGE1.CR.pIX cell line were completed so far. Phase I clinical trial revealed that immunization with MVA-NP + M1 resulted in a rapid T-cell response across age groups, which was maintained at levels above baseline responses over the course of a year [237]. In the group aged 65 years and above, MVA-NP + M1 was able to boost pre-existing levels of specific T-cells for up to at least 6 months [238]. Although there was a trend towards the vaccine group having improved outcomes after intranasal influenza challenge (less laboratory confirmed cases with less pronounced symptoms, reduction number of days of viral shedding), statistical significance was not reached ([239]; https://www.clinicaltrialsregister.eu/ctr-search/trial/2009-010334-21/results, accessed on 3 February 2022).

Modification of vaccination strategy was made by a combination of a vaccine candidate MVA-NP + M1 with licensed inactivated influenza vaccine in adults 65 years and above in a randomized, participant-blinded, placebo-controlled, multi-center phase IIb efficacy study. The aim of this vaccine strategy is to boost pre-existing cross-reactive T-cell responses to highly protective levels, providing immunity to not only strain-specific influenza, but also to heterosubtypic influenza A viruses [240,241]. This trial was terminated after one season due to a change in the recommended seasonal vaccine strain. Under such circumstances only 846 of a planned 2030 participants were recruited. The study shows expected increase in the T cell response. Since this was underpowered study, there was no benefit seen in this trial to inducing higher levels of hemagglutinin-specific antibodies by adding MVA-NP + M1, and it was unable to show any association of illness outcomes with gamma interferon T cell responses.

Different viral vector was used by Dicks et al. They constructed a novel replication-deficient chimpanzee adenovirus vector expressing conserved influenza antigens NP and M1 (ChAdOx1-NP + M1) [242]. Clinical assessment of this type of vaccine demonstrated safety in trail participants with the increased T-cell response [243].

A more immunogenic version of NP was created by fusing it to the small oligomerization domain OVX313 which led to formation of the NP heptamer named OVX836 [244] which shows increased uptake by dendritic cells and immunogenicity compared with NP. Intramuscular immunization in mice with OVX836 induced strong NP-specific CD4+ and CD8+ T-cell systemic responses and established CD8+ tissue memory T cells in the lung parenchyma [244]. Phase II clinical trial with this vaccine candidate was completed in September 2020 (NCT04192500).

#### 4.1.2. RSV Vaccine Candidates Based on the NP

In spite of the high health burden, the vaccine against RSV infection is still an elusive goal. Main reasons for that are gaps in the puzzle of the immune response elicited upon RSV infection, the variability of the surface glycoproteins and the fact that infants should be vaccinated very early upon birth when immune system is still underdeveloped. Similar to influenza, natural RSV infection does not confer lifetime immunity. Major target groups for RSV vaccination are infants, elderly and immunocompromised people. Strategy is to develop vaccine applicable for indirect vaccination of infants through maternal immunization and direct vaccination of adults at high risk of developing severe complications if infected with RSV.

NP is highly conserved between human RSV A and B serotypes. Therefore, an NP-based vaccine alone or in combination with the fusion protein-based vaccine, could be one of directions to reach the effective and safe RSV vaccine.

MVA-BN-RSV is a vector-based vaccine based on the MVA-BN backbone that can enter mammalian cells and initiate viral protein expression without replicating in most mammalian cells [245]. The vaccine was designed to encode and express five RSV antigens: surface F protein and G protein of subtypes A and B, and internal conserved proteins NP and M2 protein. In this way the vaccination is mimicking natural infection and could provide a broad and long-term protection against RSV. Completed Phase I [246] and Phase II [247] clinical trials show that MVA-BN-RSV induced robust T cell responses and moderate, but consistent humoral responses were observed against A and B RSV subtypes which persisted at least 6 months and can be boosted at 12 months, without significant safety findings.

Similar promising results were published for early phase trial for chimpanzee-adenovirus-155 vector backbone vaccine encoding RSV F protein, NP, and transcription antitermination proteins (ChAd155-RSV). The trials show that ChAd155-RSV increases specific humoral and cellular response in previously exposed adults. No severe adverse effects were observed [248].

Given the extensively accepted safety and immunogenicity profile of the bacillus Calmette–Guérin (BCG) vaccine in newborns, the vaccine based on the recombinant BCG expressing NP of RSV (rBCG-N-hRSV) is intended for direct use on neonates to prevent severe hRSV infection [201]. A phase I clinical trial rBCG-N-hRSV vaccine showed that the vaccine was safe; serum IgG antibodies directed against *M. bovis* and the N-protein of RSV increased after vaccination, as well as the cellular response, consisting of IFN-γ and IL-2 production against PPD and the N-protein. Interestingly enough, the sera of volunteers immunized with the lowest dose of rBCG-N-hRSV were capable of neutralizing RSV in vitro. However, it is possible that the neutralization capacity of some sera from this cohort results from the natural exposure to RSV since this cohort was immunized in the middle of the RSV seasonal outbreak [249].

Ebola vaccine based on the NP that is under approval process by the FDA has already been approved by the EMA and more of this vaccine will be discussed in the following section.

### 4.2. Vaccines Based on NP as Antigen in Use

The only vaccine partly or completely based on the NP is vaccine against Ebola. Outbreaks of Ebola are causing devastating consequences with >40% lethal cases. This has urged the development of effective and widely available Ebola vaccine. The vaccine was approved by the EMA in 2020 and it is under the approval process by the FDA (NCT04152486). The vaccine consists of two heterologous components given eight weeks apart [250]. The component used for prime vaccination is a replication-deficient adenovirus type 26 vector-based vaccine (Ad26.ZEBOV, Zabdeno), expressing Zaire Ebola virus glycoprotein. The second component used for the boost is an MVA vector-based vaccine, encoding glycoproteins from Zaire Ebola virus, Sudan virus, and Marburg virus, and NP from the Tai Forest virus (MVA-BN-Filo, Mvabea). This heterologous prime/boost vaccination regimen is proved as safe, well tolerated, and immunogenic, with humoral and cellular immune responses persisting for 1 year after vaccination [250]. Since both components are replicative or non-replicative viral vector platforms being an intrinsic adjuvant, no additional adjuvant is required for effective induction of protective immunity.

### 4.3. Nucleoprotein as a Scaffold for Foreign Antigen Delivery

Nanosized self-assembled proteins are used as platforms in various biomedical implementations, including the development of new vaccine candidates [251].

Nanovaccines, having self-assembled proteins as a scaffold, are showing numerous advantages. The key steps in increasing immunogenicity are uptake and processing by APCs, whose activation is important for T-cell priming and activation of B-cells [252]. Pathogen-sized particles and nanoparticles are uptaken by APCs and consequently DCs are activated through a series of downstream mechanisms. DCs can cross-present antigens, so both CD4+ and CD8+ T-cells are switched on [253]. Since the adaptive immune response is mostly induced in the lymphatic system, it is crucial for vaccines to enter lymphoid organs. The lymph vessels are 10–60 μm in diameter, so molecules from 20 to 200 nm can easily enter, and antigens can directly interact with B cells, while larger nanoparticles from 100 to 500 nm are carried by specialized cells [252]. The ability to present multiple antigens on the particle surface, use of positively charged particles and hydrophobic clusters also have an impact on increasing immunogenicity. Nanorings and nanoparticles, used in vaccination nanotechnology, resemble viral capsid structure, while larger assemblies, nanofilaments and nanotubes have the same conformation as some pathogen structures, e.g., pili, flagella and helical viral capsids [253].

NP from RSV forms a ring built from 10–11 monomers and it was used as a scaffold for influenza virus vaccine [254]. Since M2e alone is only weakly immunogenic, a conservative ectodomain of influenza virus transmembrane protein was anchored onto NP of RSV. In this study, both N-M2e and N-3M2e (three copies of epitope linked to N-protein) were expressed and purified in *E. coli*. N-M2e and N-3M2e showed stronger immune responses compared to M2e and 3M2e alone. As expected, induced anti-M2e antibodies were not neutralizing, as was the case after using conventional influenza vaccine. However, a complete virus elimination in mice infected with influenza virus after N-3M2e injection was shown. Importantly, in this study was confirmed that preexisting immunity against NP did not affect immune response against this type of vaccine.

Measles RNP was fused to circumsporozoite protein (CS) from *Plasmodium berghei*, which is present on Plasmodium surface and used as an antigen in malaria vaccine candidates. Fusion protein was expressed in Pichia pastoris and observed as a filamentous structure in yeast cytoplasm. Mice susceptible to *P. berghei* infection were immunized with whole yeasts expressing the fusion protein. A decrease in parasitemia in mice was observed [255]. In the next study, mice were immunized with clear lysate retaining recombinant N-PbCS. Yeast lysates were shown to have adjuvating properties. Additionally, PbCS fused to measles virus RNPs induced better antibody response compared with non-multimerized PbCs. Since only multimerized PbCS could cause the delay in parasitemia, RSV RNPs from yeast lysate have a promising potential as carriers for the malaria vaccine and, moreover, as a platform for the delivery of subunit vaccines from different pathogens [256]. The development of veterinary vaccines has also recognized the benefits of the NP nanoplatform. Mebatsion et al. localized a conserved B-cell immunodominant epitope (IDE) of the Newcastle disease virus (NDV) NP spanning aa 447–455 and successfully generated a recombinant NDV lacking the IDE by reverse genetics. Then they inserted a B-cell epitope of S2 glycoprotein of murine hepatitis virus (MHV) instead of the conserved IDE of NDV NP. Chickens immunized with the hybrid recombinant NDV produced specific antibodies against the S2 glycoprotein of MHV [257].

So, the use of NP as a vaccine carrier due to the self-assembling properties and the size of multimeric forms could be a new direction in a successful vaccine design.

## 5. Conclusions

In spite of the effort that has been invested in numerous studies and trials, only a few vaccines against NSVs are available indicating the need for alternative approaches when developing the new vaccines. This review revisited a large body of literature available on a highly conserved NP showing versatile function of this protein in the life cycle of the NSVs and identifying NP as a valuable target for the advancing vaccine design and target of inhibitory drugs.

In spite of the abundance of the NP in the virion or infected cell and the overwhelming antibody response to this protein, the interest in the complete understanding of the adaptive immune response to this protein is not proportional. NP-based vaccines are considered as T cell-directed vaccine and clinical trials are focused on the T cell response. At the same time, NP-specific humoral response is mostly neglected. Sporadic studies on the role of humoral response to NP in protection against NSVs have merely scratched the surface of the underlying mechanism. However, these studies show that this arm of immune response contributes to resolving the infection.

To conclude, the rational design of the vaccine against NSVs warrants better understanding of the immunobiology of each individual viral protein, and co-operation of cellular and humoral immunity against them. These fragmented views indicate NP as a potent vaccine target, either as a stand-alone vaccine or as a complementing component as has been already used for Ebola vaccine strategy.

## Figures and Tables

**Table 1:** CD4+ T-cell epitopes of the measles virus.

Location	Sequence	HLA Antigen ^1^	Reference
271–290	LTIKFGIETMYPALGLHEFA	n.d.	[128]
367–386	EMVRRSAGKVSSTLASELGI	n.d.	
400–420	TTEDKISRAVGPRQAQVSFL	n.d.	
483–502	QDPQDSRRSAEPLLRLQAMA	n.d.	
185–199	PDTAADSELRRWIKY	HLA-DRB1*1103	[130]
321–340	QNKFSAGSYPLLWSYAMGVG	n.d.	[129]
331–350	LLWSYAMGVGVELENSMGGL	n.d.	
372–385	SAGKVSSTLASELG	HLA-DRB1*0301	[131]

^1^ n.d.—not defined.

**Table 2:** Cytotoxic T-cell epitopes of the Hantaan virus.

Location	Sequence	HLA Antigen ^1^	Cross-Reactivity to Distantly Related Viruses ^1^	Reference
12–20	NAHEGQLVI	HLA-B51	yes	[152]
129–137	FVVPILLKA	HLA-A2	yes	[153]
131–139	VPILLKALY	HLA-B35	yes	[153]
167–175	DVNGIRKPK	HLA-A33	yes	[153]
197–205	RYRTAVCGL	HLA-A11	yes	[154]
245–253	KLLPDTAAV	HLA-A24	yes	[154]
247–255	LPDTAAVSL	HLA-B35	no	[153]
258–266	GPATNRDYL	HLA-B7	yes	[154]
277–285	ETKESKAIR	HLA-A33	no	[153]
301–315	SPSSIWVFAGAPDRC	n.d.	n.d.	[154]
334–342	ILQDMRNTI	HLA-A2.1	yes	[155]
355–369	LRKKSSFYQSYLRRT	n.d.	n.d.	[154]
415–429	DVKVKEISNQEPLKL	n.d.	n.d.	[154]
421–429	ISNQEPLKL	HLA-A1	yes	[152]

^1^ n.d.—not defined.

**Table 4:** Clinical trials for novel vaccines based on the NP against human NSVs in Phases 1–3 listed by the FDA (https://www.clinicaltrials.gov, accessed on 1 February 2022) or EMA (https://www.clinicaltrialsregister.eu, accessed on 3 February 2022) by 1 February 2022.

Medical Condition	Clinical Trial Identifier (Regulatory Agency)	Vaccine Type	Phase (Status)	Sponsor
influenza	2009-010334-21 (EMA)	MVA * encoding NP and M1 proteins (MVA-NP + M1)	IIa (completed in 2010)	University of Oxford
	NCT00993083 (FDA)		II (completed in 2010)	
	NCT01818362 (FDA)	chimpanzee adenovirus AdOx1 encoding NP and M1 (ChAdOx1 NP + M1)	I (completed in 2015)	
	2017-001103-77 (EMA)	seasonal inactivated influenza vaccine in combination with MVA-NP + M1	IIb (completed in 2018)	Vaccitech Limited
	NCT03300362 (FDA)		IIb (completed in 2018)	
	2021-002535-39 (EMA)	oligomerization domain OVX313 fused to NP which formed the NP heptamer (OVX836)	IIb (ongoing)	Osivax S.A.S
	NCT03594890 (FDA)		I (completed in 2019)	
RSV	2017-004582-27 (EMA)	MVA * encoding RSV antigens F, G (of subtypes A and B), NP and M2 (MVA-BN-RSV)	IIa (completed in 2019)	Bavarian Nordic
	NCT04752644 (FDA)		II (ongoing)	
	2018-000431-27 (EMA)	chimpanzee adenovirus Ad155 encoding F, NP and M2 proteins (ChAd155-RSV)	I/II (completed in 2021)	GlaxoSmithKline Biologicals
	NCT02491463 (FDA)		I (completed in 2017)	
	NCT03213405 (FDA)	BCG ** encoding RSV NP (rBCG-N-hRSV)	I (completed in 2018)	UC Chile
Ebola	NCT04152486	MVA * encoding glycoproteins of Zaire ebolavirus, Sudan ebolavirus and Marburg Marburgvirus, and NP of Taï Forest ebolavirus (MVA-BN-Filo)	III (ongoing)	London School of Hygiene and Tropical Medicine

* MVA—modified vaccinia virus Ankara strain; ** BCG—Bacillus Calmette–Guérin.

## Data Availability

Not applicable.
